# What Are the Best Parents for Hybrid Progeny? An Investigation into the Human Pathogenic Fungus *Cryptococcus*

**DOI:** 10.3390/jof7040299

**Published:** 2021-04-15

**Authors:** Man You, Jianping Xu

**Affiliations:** Department of Biology, McMaster University, Hamilton, ON L8S 4K1, Canada; youm1@mcmaster.ca

**Keywords:** *Cryptococcus* hybrids, phenotypic variation, genotypic diversity, better-parent heterosis, transgressive segregation

## Abstract

Hybridization between more divergent organisms is likely to generate progeny with more novel genetic interactions and genetic variations. However, the relationship between parental genetic divergence and progeny phenotypic variation remains largely unknown. Here, using strains of the human pathogenic *Cryptococcus*, we investigated the patterns of such a relationship. Twenty-two strains with up to 15% sequence divergence were mated. Progeny were genotyped at 16 loci. Parental strains and their progeny were phenotyped for growth ability at two temperatures, melanin production at seven conditions, and susceptibility to the antifungal drug fluconazole. We observed three patterns of relationships between parents and progeny for each phenotypic trait, including (i) similar to one of the parents, (ii) intermediate between the parents, and (iii) outside the parental phenotypic range. We found that as genetic distance increases between parental strains, progeny showed increased fluconazole resistance and growth at 37 °C but decreased melanin production under various oxidative and nitrosative stresses. Our findings demonstrate that, depending on the traits, both evolutionarily more similar strains and more divergent strains may be better parents to generate progeny with hybrid vigor. Together, the results indicate the enormous potential of *Cryptococcus* hybrids in their evolution and adaptation to diverse conditions.

## 1. Introduction

Hybridization refers to crosses between two closely related species or divergent populations within a species. Natural hybridization has been reported in most groups of eukaryotes. A diversity of phenotypes has been observed for hybrids, including heterosis (hybrid vigor), outbreeding depression, and intermediate between parents [1,2,3,4]. At present, despite our long history of studying hybrids and hybridization, the mechanisms for hybrid offspring phenotype variation remain largely unknown. A longstanding and unsolved issue is the relationship between parental population divergence and hybrid progeny phenotype. In 1936, East proposed that as the genetic distance between parental populations increases, heterosis should become more prevalent [5]. However, there has been limited critical testing of this intriguing hypothesis.

The human pathogenic *Cryptococcus* (HPC) represents an ideal group of organisms to study this hypothesis and investigate the relationships between the genetic divergence of parental strains and offspring phenotypes. HPC consists of multiple evolutionary divergent lineages that have been classified into different species complexes, species, varieties, serotypes, and molecular types [6,7]. For example, there are two species complexes within HPC, the *Cryptococcus neoformans* species complex (CNSC) and the *Cryptococcus gattii* species complex (CGSC), corresponding to an estimated divergence time of ~80–100 million years. Each of the two species complexes contains multiple divergent lineages. Importantly, these lineages show a range of nucleotide sequence divergence at the whole genome level, ranging from 2% to ~15% [6,8,9,10,11].

HPC has several virulence factors that play crucial roles in the survival and proliferation within the hosts [12]. The three essential virulence factors are the synthesis of melanin, the formation of a polysaccharide capsule, and the ability to grow at mammalian body temperature (37 °C). Melanin has antiphagocytic and antioxidant activities, protecting cryptococcal cells against environmental stressors (e.g., ultraviolet (UV) irradiation and high temperature) and antifungal agents (e.g., amphotericin B) [13,14]. The polysaccharide capsule protects the cells from being phagocytized by host phagocytic cells and allows them to evade the host immune system attack [15,16]. Combined with the ability to grow at 37 °C, these virulence-related traits allow *Cryptococcus* strains to be a pathogen and thrive in human and animal hosts.

Apart from hybrids, strains of the human pathogenic *Cryptococcus* are generally haploid. They exist in one of the two mating types, *MAT***α** and *MAT***a**, with most environmental and clinical strains belonging to *MAT***α** [17,18,19]. Haploid cells typically propagate asexually by budding until strains of the opposite mating types (**a**-**α**) or even the same mating type (**α**-**α**) encounter each other to initiate mating and sexual reproduction [20]. Sexual reproduction can give rise to the generation of diverse genotypes via recombination. This diversity and the resulting phenotypic variation could be key factors in promoting adaptation to changing environments. Evidence of mating and recombination has been reported in the human pathogenic *Cryptococcus* [18,21,22,23,24]. Although sexual reproduction is typically rare except in a few regions (e.g., Botswana and Brazil) where it is relatively frequent, evidence of both **α**-**α** and **a**-**α** matings generating diploid/aneuploid hybrids has been found [23,25,26,27,28,29,30,31,32]. Serotype AD hybrids, derived from mating between the VNI (serotype A) and VNIV lineages (serotype D) within CNSC, have been reported in environmental and clinical samples [23,28,33]. However, hybrids between CGSC and CNSC lineages have only been reported from clinical settings and the laboratory [34,35,36,37]. Of all reported hybrid populations, serotype AD hybrids are the most common, with a prevalence of 18% in Europe and 6% in the United States [38]. Among the hybrids, there are increasing reports showing evidence of hybrid vigor and transgressive segregation [1,23,39,40]. For example, serotype AD hybrids are often more resistant to UV irradiation and antifungal drugs than their parental strains, as well as more thermotolerant.

In this study, the aim was to address the following question: What are the best parents for hybrid progeny in the human pathogenic *Cryptococcus*? To broadly address this question, we selected 22 parental strains representing six evolutionarily distinct lineages of this fungus to construct crosses. We investigated the effects of parental phenotypic variations on progeny phenotypic variations and the relationship between parental strain sequence divergence and progeny phenotypes. A number of phenotypes were assayed, including growth at 30 °C and 37 °C, melanin production under various oxidative and nitrosative stresses, and susceptibility to the antifungal drug fluconazole. We observed various degrees of phenotypic variation and phenotypic plasticity among the progeny. We also identified different relationships between parental genetic divergence and progeny phenotypes among the measured traits.

## 2. Materials and Methods

### 2.1. Parental Strains and Progeny

Twenty-two *Cryptococcus* strains were used as parental strains in this study (Table 1). There were six VGI strains (two *MAT***a** and four *MAT***α**), four VGII strains (one *MAT***a** and three *MAT***α**), six VGIII strains (three *MAT***a** and three *MAT***α**), one VGIV strain (*MAT***α**), three VNI strains (one *MAT***a** and two *MAT***α**), and two VNIV strains (one *MAT***a** and one *MAT***α**). These *MAT***a** strains and *MAT***α** strains were mated on V8 agar, following the protocol described in You and Xu [37]. After two to six weeks of incubation at room temperature (~23 °C), hyphae started to form. To collect progeny, once formed, hyphae without parental cells were streaking out on new yeast extract–peptone–dextrose (YEPD) agar plates and incubated at 37 °C for three days. The 37 °C temperature was used to select diploid yeast cells from dikaryotic hyphae. Then, all single yeast colonies on each plate were picked and purified. The mating types of these colonies were determined by using the primers of *STE20***a** and *STE12***α** genes. Only those containing both mating types were selected for further analyses in this study.

Genetic distance between mating partners was calculated to estimate the parental genetic divergence, based on five gene loci (*GPD1*, *LAC1*, *PLB1*, *URA5*, and *IGS1*). All gene fragments were amplified using the polymerase chain reaction (PCR) conditions described by Meyer et al. [41]. Sequences were obtained from GenBank or sequencing. The phylogenetic analysis, using the neighbor-joining method with 1000 bootstrap replicates, was performed to compute the genetic distances between mating partners in MEGA 7.0 [42]. Details about the primers and PCR conditions are listed in Table 2.

### 2.2. Ploidy Analyses

The ploidy levels of both parents and progeny were determined by fluorescence-activated cell sorting (FACS), similar to the protocol used by Skosireva et al. [43]. Briefly, cells grown overnight were harvested from YEPD agar medium (~10^7^ cells/mL), washed once in phosphate-buffered saline (PBS), and then fixed in 1mL of 70% ethanol at 4 °C for at least 6 h. Fixed cells were washed once in NS buffer, then stained with 5 µL of propidium iodide (10 mg/mL) in 180 µL NS buffer adding 20 µL of RNaseA (10 mg/mL) and incubated with agitation for 3 h at room temperature or overnight at 4 °C 50 µL of stained cells were diluted into 2 mL of 50 mM Tris-HCl (pH 8.0) and sonicated for 10s. Flow cytometry was performed on a Becton–Dickinson LSR II model with ~10^4^ cells. Data were analyzed and visualized by ModFit LT 5.0 (Verity Software House). Parental strain JEC21 was used as a haploid control, and D15 (RAS strain) was used as a diploid control [44].

### 2.3. Polymerase Chain Reaction-Restriction Length Polymorphism (PCR-RFLP) Genotyping

**Figure 1:**
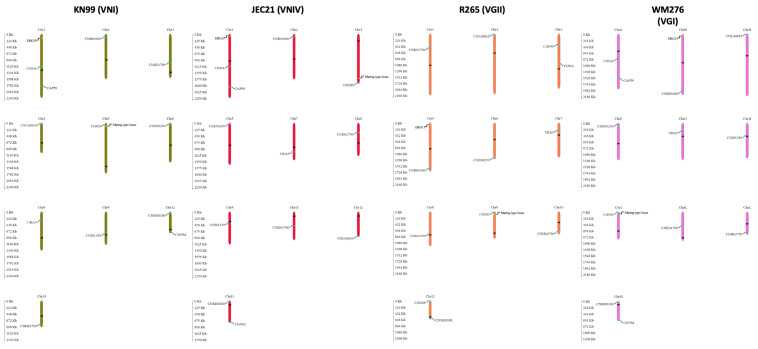
Chromosomal locations of the fourteen polymerase chain reaction-restriction length polymorphism (PCR-RFLP) markers that were used for progeny genotyping in this study. These markers were located across 10 chromosomes (out of 14) on the four reference genomes of strains, KN99 (VNI lineage), JEC21 (VNIV lineage), R265 (VGII lineage), and WM276 (VGI lineage). Dark text indicates the location of centromeres.

Fourteen nuclear markers and two mitochondrial markers (*ND2* and *ND4*) were used for genotyping. The 14 nuclear markers are located on 10 of 14 chromosomes, including *CAP59*, *CGNA*, *ERG11*, *CNB00360*, *CNK01700*, *CNL06810*, *CGND*, *MAT* locus (*STE12***α** and *STE20***a**), *CNE00250*, *URA5*, *CNI01350*, *CNM00180*, *CGNM2*, and *CNH02750*. Except for the *MAT* locus, the remaining 15 markers were used for polymerase chain reaction-restriction length polymorphism (PCR-RFLP) genotyping, distinguishing the parental strains for each successful cross. The choice of markers to represent the chromosomes was based on the genome annotation of the model strain JEC21 (of the VNIV lineage). These PCR-RFLP markers were either obtained from the previous studies [45,46,47] or designed using Prifi [48] based on the whole genome sequences of KN99 (VNI lineage), JEC21 (VNIV lineage), R265 (VGII lineage), and WM276 (VGI lineage). PCR products were analyzed on 1.0% agarose gels. All restriction digestions were performed following the manufacturer′s instructions (NEB, UK) and separated on 2.0% agarose gels at 80 V for 2 h. Figure 1 shows the locations of the twelve nuclear markers across chromosomes. The information on the markers and restriction enzymes used for PCR-RFLP genotyping is listed in Table 2.

### 2.4. Phenotype Assays

Parental strains and progeny were grown on YEPD agar for two days at 30 °C. Fresh cells were suspended in sterile water for the following phenotyping experiments.

For growth assay, the cell density was adjusted to ~10^6^ cells/mL in medium Roswell Park Memorial Institute (RPMI) 1640. 200 µL of each strain, with three replicates, was then inoculated in 96-well microplates and incubated at 30 °C or 37 °C for three days. The growth potential was determined by spectrophotometer using optical density (OD) of 600 nm.

For melanin assay, cell density was adjusted to ~10^6^ cells/mL in sterile water. Oxidative stresses caused by reactive oxygen species (ROS) and nitrosative stresses caused by reactive nitrogen species (RNS) were generated by adding hydrogen peroxide (H_2_O_2_) and sodium nitrite (NaNO_2_) to the caffeic acid agar, respectively, with three concentrations (0.25 mM, 0.5 mM, and 1 mM) of each. The regular caffeic-acid agar [49] without any added stress was used as the control. 5 µL of each cell suspension for each strain, with four replicates, was spotted onto the agar plates and incubated at 30 °C for seven days. Melanin production was approximated by light reflection using ImageJ [50]. Parental strains and their progeny were plated on the same plates to mitigate the potential batch effects. Additionally, JEC21 (VNIV lineage) was used as a reference on each plate for data standardization.

For antifungal susceptibility assay, concentrations of 0 µg/mL, 0.5 µg/mL, 1 µg/mL, 2 µg/mL, 4 µg/mL, 8 µg/mL, 16 µg/mL, 32 µg/mL, 64 µg/mL, and 128 µg/mL of fluconazole were evaluated, following the M27-A3 guidelines (the Clinical and Laboratory Standards Institute, CLSI) [51]. The adjusted cells were inoculated in 96-well plates and incubated for 72h at 35 °C (the CLSI recommended temperature) and 37 °C (the mammalian body temperature), respectively. The minimum inhibitory concentration (MIC) was determined in the treated samples.

### 2.5. Statistical Analysis

The coefficient of variation (CV) was used as an index to determine (i) phenotypic variation between parental strain pairs and among progeny from each cross; and (ii) phenotypic plasticity of each strain across environments for each trait. We analyzed the amounts of phenotypic variations among progeny from each cross and compared them with their parents under the same experimental condition, using CV_s_ values. A higher CV_s_ value indicates a greater phenotypic variation. We calculated two CV_i_ values for individual progeny and each parental strain, one for growth under two temperature conditions and the other for melanin production under seven conditions, and compared them with their parental strains. A high CV_i_ value indicates high phenotypic plasticity in the specific trait for the strain, while a low CV_i_ value suggests that the trait is relatively stably expressed under diverse conditions for the strain. The details of CV_s_ and CV_i_ are shown in Table S1. The Pearson correlation tests were used to determine the potential relationships between parental genetic distance and the phenotypic values of progeny under each environmental condition.

The better-parent heterosis (BPH) was calculated to determine progeny performance as compared to their parents. BPH is the percentage of progeny that had a higher phenotypic value than the high-phenotype value parent. In addition, the Pearson correlation tests were performed to determine its potential relationship with parental genetic distance. Furthermore, evidence for transgressive segregation was evaluated for progeny by comparing their phenotypic values with the phenotype range of their respective parents for each of the traits. Trait values that are two standard deviations higher than the high-value parent or two standard deviations lower than the low-value parent are referred to as positive and negative transgressive segregants, respectively.

All statistical analyses and visualization of data were performed using R (version 4.0.3) [52]. The progeny multilocus genotypes were determined using the R package “poppr” [53]. The relationships between parental genetic distances and progeny phenotypes were estimated by performing a generalized linear model using the R package “lme4” and displayed and predicted by effect plots using the R package “effects” [54,55]. The Pearson correlation tests were used to determine the correlation between parental genetic distance and progeny phenotype, phenotypic plasticity, and BPH rates. All figures were visualized using the R package “ggplot2” [56].

## 3. Results

We constructed genetic crosses and collected progeny. The ploidy levels of all progeny were determined by FACS. For all parental strains and their progeny, we obtained the quantitative phenotype values of their growth ability, melanin production, and fluconazole susceptibility. Based on the quantitative data, several analyses were performed. Below we describe the results of our genotypic and phenotypic assays of the progeny.

### 3.1. Progeny Collection and Ploidy Analyses

In our previous analyses, we found that strains of VGIII lineage were generally more fertile than those of other lineages. Thus, we constructed crosses using at least one VGIII strain in each cross. By using 22 parental strains, 58 genetic crosses were attempted and 22 of them were successful. These 22 successful crosses consisted of six (out of nine attempted) intra-VGIII crosses, three (of 18 attempted) inter-lineage VGIxVGIII crosses, two (of 13 attempted) inter-lineage VGIIxVGIII crosses, one (of three attempted) inter-lineage VGIVxVGIII cross, five (of nine attempted) inter-lineage VNIxVGIII crosses, and five (of six attempted) inter-lineage VNIVxVGIII crosses. Details of successful crosses and their progeny are listed in Table 3.

**Figure 2:**
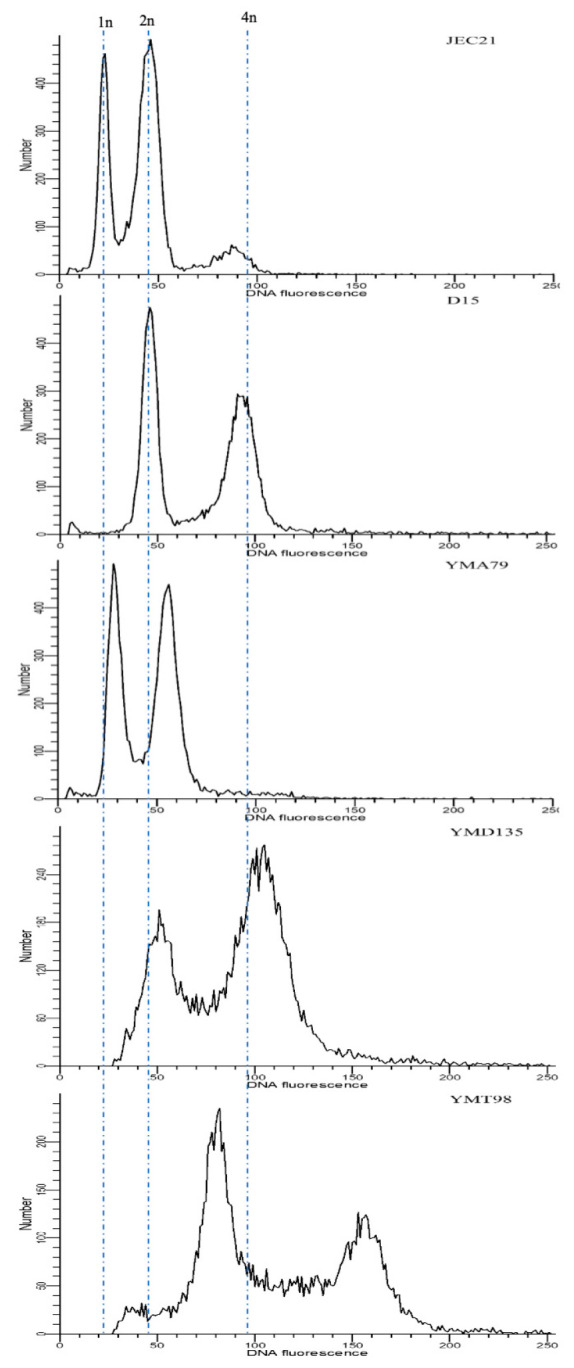
Three different ploidy levels were observed among progeny. Fluorescence-activated cell sorting (FACS) profiles of haploid control JEC21 (1n), diploid control D14 (2n), likely aneuploid progeny YMA79 (between 1n and 2n), likely diploid progeny YMD135 (2n), and likely triploid progeny YMT98 (between 2n and 4n) are shown here. 1n, 2n, and 4n indicate nuclear DNA content. The *x*-axis shows the relative fluorescence intensity of DNA content, and the *y*-axis represents the number of the counted cells of each fluorescence intensity category.

Previous studies have shown that the human pathogenic *Cryptococcus* hybrids are often diploid/aneuploid and are heterozygous at the mating-type locus. Thus, to standardize our comparison, we selected only progeny that contained mating-type genes from both parents for downstream analyses. From the 22 crosses, 55 progeny were collected for further study by confirming the heterozygosity at the *MAT* locus. FACS analyses revealed that 34 out of 55 progeny (~62%) were likely diploid, with twice the amount of DNA of the haploid parental strains and similar to the diploid control. Nineteen progeny (~35%) had FACS profiles intermediate between haploidy and diploidy, consistent with aneuploidy. The remaining two progeny had ploidy levels higher than the diploid reference strain, with profiles consistent with triploidy. Figure 2 shows the representative FACS profiles of the obtained progeny. The ploidy estimates of all 55 progeny are presented in Table 3.

### 3.2. Genotypic Diversity and Variable mtDNA Inheritance

Aside from the *MAT* locus, we analyzed 13 nuclear and two mitochondrial markers using PCR-RFLP for progeny genotyping. However, due to the high sequence similarities among the VGIII parental strains, the 18 progeny from the six intra-VGIII lineage crosses could not be distinguished from each other using these PCR-RFLP markers. For the remaining 37 hybrid progeny from the 16 inter-lineage crosses, their multilocus genotypes were determined.

All 37 hybrids were heterozygous at *ERG11* and *MAT* locus, while they were homozygous at one locus *CAP59* (Table S1). Most hybrids were heterozygous at the remaining 11 nuclear loci. Across the 14 nuclear loci, the rates of heterozygosity among the hybrids ranged from ~57% to ~93% (Table S1). We found that the parental genetic distance was significantly negatively correlated with the frequency of progeny that were homozygous for alleles from the *MAT***a** parents (*r* = −0.4, *p* = 0.014). That is, with an increasing genetic distance between parental strains, there was a significant decrease in the proportion of progeny that were homozygous for alleles from the *MAT***a** parents. The 37 hybrids were assigned to 22 unique multilocus genotypes (Table 3). Among these hybrids, 11 hybrids from four crosses had identical genotypes at the assayed loci with their siblings, whereas 26 hybrids from another nine crosses had different genotypes.

Analyses of the two mitochondrial markers identified three mitochondrial inheritance patterns among the 37 hybrids (Table S1). They included that 20 hybrids inherited mtDNA from the *MAT***a** parents (~54%), seven inherited from the *MAT***α** parents (~19%), and ten hybrids had recombinant mitochondrial genotypes (~27%, with each having a combination of alleles from the *MAT***α** and *MAT***a** parents for the two different mitochondrial marker genes). Interestingly, all recombinant mitochondrial genotypes were found in progeny derived from the inter-lineage crosses between strains of the two species complexes, CNSC and CGSC. Of the 10 hybrids with recombinant mitochondria, all had the *ND2* allele from the *MAT***α** parents and the *ND4* allele from the *MAT***a** parents.

### 3.3. Growth at 30 °C and 37 °C

#### 3.3.1. Phenotypic Variation

As shown in Table S2, parental strains showed a range of growth ability at both 30 °C and 37 °C. CV_s_ values of parental strains ranged from 0.011 to 0.664 at 30 °C and from 0.004 to 0.356 at 37 °C. The big ranges were found for both evolutionarily similar and divergent strain pairs. For example, at 30 °C, divergent parental pair JEC21 (VNIV lineage) and JF109 (VGIII lineage) grew similarly (CV_s_ = 0.011) while another divergent pair KN99**α** (VNI lineage) and B4546 (VGIII lineage) showing a CV_s_ value of 0.664. An intra-VGIII parental strain pair B4546 and B4544 showed a notable difference at 30 °C (CV_s_ = 0.650) while little difference at 37 °C (CV_s_ = 0.017). The wide range of parental phenotype variations enabled us to examine the effects of parental strain divergence on progeny phenotype relative to such variations.

We also found a range of CV_s_ values among progeny, ranging from 0.022 to 0.246 at 30 °C and from 0.013 to 0.348 at 37 °C (Table S2). The progeny from the intra-VGIII cross JF109xJF101 showed the greatest CV_s_ at 30 °C (CV_s_ = 0.246). At 37 °C, the progeny from KN99axJF101 (VNIxVGIII) showed the most variation (CV_s_ = 0.348). The progeny from JEC20xJF101 (VNIVxVGIII) had relatively low CV_s_ values at both temperatures (CV_s_ values 0.053 and 0.013, respectively). Of the 19 crosses with each having two or more progeny, the progeny of five crosses (~26%, 5/19) showed a greater CV_s_ at 30 °C while the progeny from nine crosses (~47%, 9/19) showed higher CV_s_ at 37 °C than those of their respective parental pairs (highlighted in Table S2). Progeny of two inter-lineage crosses, CDC15xJF109 (VNIxVGIII) and JEC21xATCC32608 (VNIVxVGIII), displayed greater differences in growth at both temperatures than those between parental strains.

Between the two temperatures, the progeny showed overall greater growth differences than parental strains, mainly due to an overall higher growth reduction of most progeny at 37 °C (*p* < 0.001). In contrast, the parental strains remained relatively stable at the two temperatures (*p* = 0.301).

Interestingly, the observed phenotypic variation between parents was significantly negatively associated with that between progeny at 37 °C (r = −0.46, *p* = 0.049), while not at 30 °C (r = −0.19, *p* = 0.435). In addition, there was a significant negative correlation between ploidy levels and growth ability at both 30 °C (r = −0.32, *p* = 0.008) and 37 °C (r = −0.47, *p* < 0.001) when both parental strains and progeny were included in the analyses. Similarly, parental genetic distance was significantly negatively correlated with the relative growth rates of progeny at both 30 °C (r = −0.57, *p* < 0.001) and 37 °C (r = −0.46, *p* < 0.001). However, when progeny from the intra-VGIII crosses were excluded, parental genetic distance was found significantly positively correlated with progeny growth at 37 °C (r = 0.34, *p* = 0.037) while not significantly correlated at 30 °C (r = −0.21, *p* = 0.220).

#### 3.3.2. Better-Parent Heterosis and Transgressive Segregation

**Figure 3:**
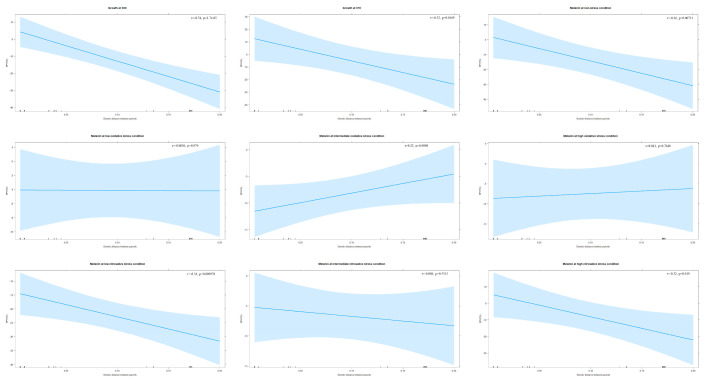
Effects of parental genetic divergence on %BPH (better-parent heterosis) in growth (30 °C and 37 °C) and melanin synthesis (non-stress, low oxidative stress, intermediate oxidative stress, high oxidative stress, low nitrosative stress, intermediate nitrosative stress, and high nitrosative stress). The *x*-axis shows the genetic distance between parental pairs. The *y*-axis represents the percentage of BPH.

Of the 55 progeny, 14 (~26%) at 30 °C and 21 (~38%) at 37 °C displayed BPH (Table S3). Ten progeny showed BPH at both temperatures, including three progeny (YMA62, YMA63, and YMA64) from JF109xB4544 (intra-VGIII). Significantly, the genetic distance between parents was negatively associated with BPH rates of progeny at both 30 °C (r = −0.54, *p* < 0.001) and 37 °C (r = −0.32, *p* = 0.017) (Figure 3). Also, transgressive segregation in both positive and negative directions was observed at both temperatures (Table S3). At 30 °C, five progeny (~9%) showed positive transgressive phenotypes, whereas three progeny (~6%) had negative transgressive phenotypes. At 37 °C, 14 progeny (~26%) displayed positive transgressive segregation, while nine (~16%) exhibited negative transgressive segregation.

#### 3.3.3. Phenotypic Plasticity

We found a range of CV_i_ values in growth for both parental strains and their progeny (Table S2). CV_i_ values ranged from 0.065 to 0.488 among parental strains and from 0.055 to 0.553 among progeny. The range of variation was not related to specific lineages among parental strains. For example, JF101 (VGIII lineage) had a very low CV_i_ value of 0.064, whereas B4544 (VGIII lineage) had the highest CV_i_ value of 0.488. Among progeny, the overall highest CV_i_ values were found from B4495xB4544 (VGIxVGIII) where all three progeny had CV_i_ values ranged from 0.534 to 0.553. In contrast, two progeny YMA162 and YMD165 from CDC15xJF109 (VNIxVGIII) had the lowest CV_i_ values (0.055 and 0.082, respectively).

When comparing each progeny with its respective parents, we found several patterns. Some progeny showed more variation in growth than their parents. However, others showed variations either in-between the parents or less than both parents. Overall, 20 of the 55 progeny (~36%) had higher CV_i_ values than both parents (highlighted in Table S2), indicating that they displayed greater plasticity than their parents in growth. For the remaining 35 progeny, 14 progeny had lower CV_i_ values than both parents, while 21 had intermediate CV_i_ values between their parents. Among the 22 crosses, we found: all progeny of four crosses had greater CV_i_ values than their parental strains; all progeny of four other crosses had lower CV_i_ values than both parents; the remaining 14 crosses had progeny as intermediates of the parental strains or a mixture of patterns. However, there was no correlation between parental genetic distance and progeny phenotypic plasticity in growth (r = −0.02, *p* = 0.8712). Our data showed a variety of growth patterns for the progeny when compared to their parents when exposed to different temperatures.

### 3.4. Melanin Production at Various Environmental Conditions

#### 3.4.1. Melanin at the Non-Stress Condition

##### Phenotypic Variation

The CV_s_ values between parents varied from 0.010 to 0.567 (Table S2). Both genetically divergent and closely related parental pairs had a wide range of CV_s_ values. For example, among intra-VGIII crosses, the parental pair ATCC32608 and JF101 showed little difference (CV_s_ = 0.010), while the parental pair ATCC32608 and B4544 displayed big differences (CV_s_ = 0.477). Similarly, among inter-lineage crosses, parental pair KN99a (VNI lineage) and JF101 (VGIII lineage) produced comparable melanin (CV_s_ = 0.047), whereas parental pair JEC20 (VNIV lineage) and B4544 (VGIII lineage) produced notably different amount of melanin (CV_s_ = 0.567).

Similarly, progeny also had a range of CV_s_ values, from 0.019 to 0.554 (Table S2). For example, the progeny from the inter-lineage VGIxVGIII cross B4545xJF101 showed the most difference in melanin (CV_s_ = 0.554), while progeny of the inter-lineage VNIVxVGIII cross JEC20xB4544 produced the most similar amount of melanin (CV_s_ = 0.019). In total, progeny of six crosses showed greater variation in melanin than that between their parents (highlighted in Table S2). However, the observed variation between parents was not associated with that among progeny (r = 0.32, *p* = 0.182).

##### Better-Parent Heterosis and Transgressive Segregation

Of the 55 progeny, 10 progeny (~18%) showed evidence of BPH (Table S3). Eight out of these ten were from five intra-VGIII crosses and two were from two inter-lineage VGIxVGIII crosses. Significantly, parental genetic distance was negatively correlated with the BPH rates of progeny (r = −0.36, *p* = 0.007) (Figure 3). Also, transgressive segregation in both directions was observed (Table S3). Among the 55 progeny, three (~5%) displayed transgressive phenotypes in the positive direction, while four (~7%) had negative transgressive phenotypes. Surprisingly, all three progeny of KN99axJF101 (VNIxVGIII) displayed negative transgressive phenotypes. Three out of the 10 progeny with BPH showed positive transgressive segregation. Overall, our results suggest that genetically more divergent parents generated fewer progeny with BPH in melanin production at the non-stress condition.

#### 3.4.2. Melanin at Oxidative Stress Conditions

##### Phenotypic Variation

Parental strains showed a range of melanin production at the three oxidative stress levels (Table S2). CV_s_ values varied from 0.001 to 0.436 at low, from 0.001 to 0.212 at intermediate, and from 0.001 to 0.535 at high oxidative stresses. Of the 22 parental pairs, 20 (~91%) at low, 19 (~86%) at intermediate, and 16 (~73%) at high oxidative stresses showed relatively small differences with CV_s_ values less than 0.1. Low and high CV_s_ values were observed for both evolutionarily similar and divergent strain pairs, although most pairs had small CV_s_ values under three oxidative stresses. The results indicate that most parental pairs, either evolutionarily similar or divergent, produced similar amounts of melanin at oxidative stresses.

Variations among progeny were also observed under three oxidative stresses (Table S2). CV_s_ values ranged from 0.008 to 0.324 at low, from 0.003 to 0.245 at intermediate, and from 0.004 to 0.420 at high oxidative stresses. Under low oxidative stress, progeny from 17 (~89%, 17/19) crosses had CV_s_ values less than 0.1, with progeny of ATCC32608xB4544 (intra-VGIII) showing the most variation (CV_s_ = 0.324), while progeny of JF109xB4544 (intra-VGIII) showing the least difference (CV_s_ = 0.008). Interestingly, both parental strains and progeny from ATCC32608xB4544 (intra-VGIII) showed the most variation as compared to other crosses at low oxidative stress. Under intermediate stress, progeny from 17 out of 19 (~89%) crosses had CV_s_ values less than 0.1. Progeny from B4545xJF101 (VGIxVGIII) showed the highest CV_s_ of 0.245, while progeny of ATCC32608xB4544 (intra-VGIII) produced the most comparable melanin (CV_s_ = 0.003). Under high oxidative stress, progeny from 14 crosses (~74%, 14/19) had CV_s_ values less than 0.1. Progeny of CDC15xJF109 (VNIxVGIII) had the smallest CV_s_ value of 0.004, whereas progeny from B4546xB4544 (intra-VGIII) had the greatest CV_s_ value of 0.420.

Similar to parental strains, most progeny produced comparable melanin under all oxidative stresses. Of the 19 crosses with progeny having CV_s_ values, progeny of ten (~53%) crosses at low, nine (~47%) crosses at intermediate, and seven (~37%) crosses at high oxidative stresses had greater CV_s_ values than their parents (highlighted in Table S2). Interestingly, there were significant positive correlations between the observed parental variation and progeny variation at low (r = 0.79, *p* < 0.001) and high (r = 0.694, *p* = 0.001) oxidative stresses. However, no correlation was found at intermediate oxidative stress (r = 0.05, *p* = 0.835).

##### Better-Parent Heterosis and Transgressive Segregation

Of the 55 progeny, 18 (~33%) at low, five (~9%) at intermediate, and 18 (~33%) at high oxidative stresses showed BPH (Table S3). Four progeny (YMA73, YMA74, YMD85, and YMD1) displayed BPH at all three stresses. Eight progeny (~15%, 8/55) showed BPH at both low and high oxidative stresses. However, parental genetic distance was not correlated with the progeny BPH rates at any of the three tested oxidative stresses (r = 0.003, *p* = 0.979, at low stress; r = 0.23, *p* = 0.091, at intermediate stress; r = 0.04, *p* = 0.765, at high stress; Figure 3).

Transgressive segregation was also observed (highlighted in Table S3). Specifically, 11 progeny (20%, 11/55) at low, one progeny (~2%, 1/55) at intermediate, and 10 progeny (~18%, 10/55) at high oxidative stresses showed positive transgressive segregation. Two progeny, YMA73 and YMD85, exhibited positive transgressive phenotypes at all three stresses. In contrast, seven progeny (~13%, 7/55) at low, nine progeny (~16%, 9/55) at intermediate, and six progeny (~11%, 6/55) at high stresses showed negative transgressive segregation. Among these, four progeny (YMD25, YMD26, YMD27, and YMD87) from inter-lineage crosses showed negative transgressive phenotypes at all three stresses. Interestingly, YMD16 showed negative transgressive phenotypes at low and intermediate stresses but positive transgressive phenotypes at high oxidative stress. Overall, more progeny showed BPH and transgressive segregation at low and high oxidative stresses than the intermediate stress.

#### 3.4.3. Melanin at Nitrosative Stress Conditions

##### Phenotypic Variation

Parental pairs in both intra-VGIII and inter-lineage crosses varied in melanin production under nitrosative stresses (Table S2). CV_s_ values of parental pairs ranged from 0.012 to 0.368 at low and from 0.016 to 0.267 at intermediate stresses. Among the 22 crosses, 15 crosses (~68%) at low stress and 12 crosses (~55%) at intermediate stress had CV_s_ values greater than 0.1. Similarly, variations were also observed among progeny. CV_s_ values of progeny varied from 0.011 to 0.258 at low stress and from 0.003 to 0.159 at intermediate stress. Under low nitrosative stress, progeny of KN99**α**xB4546 (VNIxVGIII) produced the most similar amount of melanin (CV_s_ = 0.011), while progeny of B4545xJF101 (VGIxVGIII) showed the greatest variation (CV_s_ = 0.258). Under intermediate stress, progeny from B4545xJF101 (VGIxVGIII) showed the least differences (CV_s_ = 0.003), while progeny from JF109xJF101 (intra-VGIII) displayed the most variation (CV_s_ = 0.159). In contrast, parental pair B4495 and JF101 (VGIxVGIII) showed little variation at low stress (CV_s_ = 0.012) but notable differences at intermediate stress (CV_s_ = 0.115). Of the 19 crosses with ≥ two progeny, progeny of three crosses (~16%) had greater CV_s_ values than those of their parents at low and intermediate stresses, respectively (highlighted in Table S2). Different from parental strains, progeny from most crosses had CV_s_ values less than 0.1 at low (~79%, 15/19) and intermediate (~74%, 14/19) stresses. Additionally, no correlations were found between parental variation and progeny variation under these two conditions.

However, by contrast with low and intermediate stress conditions, eight parental strains failed to grow under high nitrosative stress. They were: VGIII strains B4546, B4544, JF109, ATCC32608, and JF101; VGI strain B4545; VGIV strain WM779; VGII strain LA55. In total, there were nine crosses involving these parental strains that neither was able to grow at high nitrosative stress. Among these nine crosses, progeny from eight crosses also all failed to grow at high nitrosative stress. Surprisingly, although neither parental strain grew at high nitrosative stress, progeny YMD85 survived. Another two progeny, YMD150 and YMD16, failed to grow, although their siblings grew. We found 14 crosses having at least one progeny that grew, in spite of one parental strain (VGIII lineage) being unable to grow. Because of the low viability and small sample size, we were able to calculate CV_s_ values of progeny for only 11 crosses that had at least two progeny growing (excluding all non-viable progeny; Table S2). Among these 11 crosses, CV_s_ values of progeny ranged from 0.011 to 0.1967. Progeny of JEC21xATCC32608 (VNIVxVGIII) showed the most variation in melanin (CV_s_ = 0.197), whereas progeny of KN99**α**xB4546 (VNIxVGIII) had little difference in melanin (CV_s_ = 0.011). Of these 11 crosses, only two crosses (~18%) had progeny with CV_s_ values greater than 0.1. Together, under all nitrosative stresses, most parental strains showed notable variations while most of their progeny produced comparable melanin.

##### Better-Parent Heterosis and Transgressive Segregation

Of the 55 progeny, five (~9%) at low, 16 (~29%) at intermediate, and seven (~13%) at high nitrosative stresses displayed BPH (Table S3). Although no progeny showed BPH at all nitrosative stresses, six progeny (~11%, 6/55) showed BPH at two stresses. For example, YMD36 and YMD111 showed BPH at both intermediate and high nitrosative stresses. There were significant negative associations between parental genetic distance and progeny BPH rates at low and high nitrosative stresses (r = −0.34, *p* = 0.010 at low; r = -0.32, *p* = 0.019 at high; Figure 3). However, there was no such correlation at intermediate nitrosative stress (r = −0.086, *p* = 0.531; Figure 3).

Additionally, transgressive segregation in both directions was observed at all nitrosative stresses (highlighted in Table S3). Among the 55 progeny, three progeny (~5%) at low stress, seven progeny (~13%) at intermediate stress, and three progeny (~5%) at high stress had positive transgressive phenotypes. Although no progeny showed positive transgressive segregation at all three nitrosative stress levels, three progeny were positive transgressive segregants: progeny YMD86 at low and high nitrosative stresses; while progeny YMD36 and YMD111 at both intermediate and high nitrosative stresses (Table S3). In contrast, five progeny (~9%, 5/55) at low and one progeny (~2%, 1/55) at intermediate stress levels displayed negative transgressive phenotypes, but none of the progeny showed negative transgressive segregation at high nitrosative stress. Overall, though the rate of progeny showing transgressive segregation was low, positive transgressive phenotypes were observed at all three tested nitrosative stresses.

#### 3.4.4. Phenotypic Plasticity

A range of CV_i_ values was observed for both parental strains and their progeny in melanin production across the tested six or seven conditions (Table S2). CV_i_ values ranged from 0.071 to 0.556 among parental strains and from 0.094 to 0.651 among progeny. Of the 22 parental strains, B4545 (VGI lineage) had the greatest CV_i_ value, while JEC21 (VNIV lineage) had the smallest CV_i_ value. Interestingly, except for JEC21, all other parental strains had CV_i_ values of >0.1, with most strains in most lineages showing a range of CV_i_ values. Among the 55 progeny, YMD85 had the highest CV_i_ value, while YMD16 had the smallest CV_i_ value. Except for YMD16, all progeny had CV_i_ values greater than 0.1. Of the 55 progeny, ten progeny (~18%) had greater CV_i_ values than that of both parents (highlighted in Table S2). Fourteen progeny (~25%, 14/55) had lower CV_i_ values than both parents, whereas another 31 progeny (~56%, 31/55) had intermediate CV_i_ values between parents. Among the 22 crosses, all three progeny of one cross had higher CV_i_ values than their parents, all progeny of two crosses had lower CV_i_ values than their parents, and progeny of the remaining 19 crosses had a mixture of lower, intermediate, and/or higher CV_i_ values (Table S2). However, parental plasticity in melanin production was not correlated with progeny plasticity (r = 0.30, *p* = 0.093). Significantly, there was a negative correlation between parental genetic distance and progeny plasticity in melanin (r = −0.54, *p* < 0.001). Overall, we found that progeny showed three types (less, intermediate, and greater) of plasticity in melanin as compared to their respective parental strains.

#### 3.4.5. Relationships between Oxidative and Nitrosative Stresses

First, we compared melanin production between oxidative stresses separately for the parental strains and the progeny population. For parental strains, melanin production at intermediate oxidative stress was significantly positively correlated with that at high oxidative stress (r = 0.75, *p* < 0.001). However, such a significant correlation was not observed between low and intermediate oxidative stresses (r = 0.23, *p* = 0.140) or between low and high oxidative stresses (r = 0.27, *p* = 0.075). For progeny, we found significantly positive correlations between low and high oxidative stresses (r = 0.34, *p* = 0.012) and between intermediate and high oxidative stresses (r = 0.36, *p* = 0.006), while not found between low and intermediate stresses (r = 0.25, *p* = 0.069).

Second, we compared melanin production between nitrosative stresses. For parental strains, there were significant positive correlations in melanin productions between low and intermediate nitrosative stresses (r = 0.43, *p* = 0.004), and between low and high nitrosative stresses (r = 0.67, *p* = 0.012). However, no correlation was found between intermediate and high nitrosative stresses (r = −0.42, *p* = 0.16). For progeny, a significant positive correlation was observed between low and intermediate nitrosative stresses (r = 0.60, *p* < 0.001), while no correlation was observed between intermediate and high stresses (r = −0.06, *p* = 0.75) or between low and high stresses (r = 0.08, *p* = 0.65).

Third, we investigated the relationships between oxidative and nitrosative stresses (Table S4). For parental strains, there were significant positive correlations between melanin production at low oxidative stress and low nitrosative stress (r = 0.32, *p* = 0.033), between high oxidative and low nitrosative stresses (r = 0.55, *p* < 0.001), and between high oxidative and intermediate nitrosative stresses (r = 0.53, *p* < 0.001). However, no correlations were found between other stresses. The results indicate that those parental strains that produced more melanin under low and/or high oxidative stresses are likely to produce more melanin at low and/or intermediate nitrosative stresses.

Similarly, we also found several correlations for progeny between oxidative and nitrosative stresses. There were significant positive correlations in melanin production between low oxidative stress and low nitrosative stress (r = 0.66, *p* < 0.001), between intermediate oxidative and low nitrosative stresses (r = 0.37, *p* = 0.006), between high oxidative and low nitrosative stresses (r = 0.47, *p* < 0.001), and between high oxidative and intermediate nitrosative stresses (r = 0.34, *p* = 0.010). However, no correlations were observed between other stresses. Our data demonstrate that those progeny that produced more melanin under any of the three oxidative stresses are likely to produce more melanin at low and/or intermediate nitrosative stress. Overall, our findings suggest, for parental strains and progeny, there were both shared and distinct mechanisms for melanin biosynthesis between oxidative and nitrosative stresses at different levels.

#### 3.4.6. Effects of Potential Factors on Melanin Production

Compared to the non-stress condition, both the parental strains and the progeny overall produced significantly less melanin at all oxidative and two lower nitrosative stresses (*p* values < 0.001; Figures S1 and S2). Among oxidative stresses, both parental strains and progeny had more melanin production on average at the intermediate oxidative stress than the other two levels. Significantly, we found that parental strains at intermediate oxidative stress produced more melanin than at high oxidative stress (*p* < 0.001), while such a significant difference was not observed in progeny. Among nitrosative stresses, for both parental strains and progeny, significant differences in melanin production were found between low and intermediate (*p* values = 0.003 and <0.001 respectively) and between low and high nitrosative stresses (*p* values = 0.01 and <0.001 respectively).

We found significant negative correlations between parental genetic distance and progeny melanin production at non-stress condition (r = −0.64, *p* < 0.001), all three oxidative stress levels (r = −0.42, *p* = 0.002 at low; r = −0.35, *p* = 0.01 at intermediate; r = −0.44, *p* < 0.001 at high), and low nitrative stress (r = −0.44, *p* < 0.001). When progeny from all intra-VGIII crosses were excluded from analyses, significant negative correlations were still found at non-stress condition (r = −0.43, *p* = 0.009) and all oxidative stresses (r = −0.55, *p* = 0.001 at low; r = −0.43, *p* = 0.009 at intermediate; r = −0.55, *p* < 0.001 at high), while no correlations were found at any nitrosative stresses. In addition, we found significant negative correlations between ploidy levels and melanin production at non-stress (r = −0.37, *p* = 0.003) and low nitrosative stress (r = −0.32, *p* = 0.009) conditions, while no correlations were found at other stresses.

Taken together, our results here illustrate that, for both parental strains and progeny, melanin production could be affected by both environmental stresses and ploidy changes. Under non-stress and oxidative stress conditions, progeny of evolutionarily more similar parental pairs produced overall more melanin than those from evolutionarily more divergent parents.

### 3.5. Susceptibility to Antifungal Drug Fluconazole

Susceptibility to fluconazole was evaluated for all parental strains and their progeny, obtaining their minimal inhibitory concentration (MIC) values (Table 3). Consistently, we obtained the same MIC values for each tested strain at 35 °C and 37 °C. The MIC values of parental strains ranged from 1 µg/mL to 32 µg/mL. Among them, two parental strains had MIC values of 32 µg/mL, four had MIC of 4 µg/mL, four had MIC of 2 µg/mL, and five had MIC of 1 µg/mL. Among strains used in this study, parental strains of CGSC had overall higher MIC values than those of CNSC.

Similarly, progeny also had MIC values ranging from 1 µg/mL to 32 µg/mL. Of the 55 progeny, 19 (~35%) had greater MIC values than both of their parents; 24 (~44%) had the same MIC values as their more resistant parents; nine (~16%) had intermediate MIC values; two (~4%) had the same MIC values as their less resistant parents; one (~2%) had a lower MIC value than both of its parents. We found a significantly positive correlation between parental genetic distance and MIC values of progeny (r = 0.28, *p* = 0.039). However, when the five progeny of CDC15 (VNI lineage) with MIC values of ≥16 µg/mL were excluded, there was no correlation between parental genetic distance and progeny MIC values (r = 0.02, *p* = 0.873).

Additionally, we standardized progeny MIC values by calculating the percentage of progeny MIC values to the mean MIC values of two parental strains. A significant positive correlation was observed between parental genetic distance and the standardized MIC values (r = 0.32, *p* = 0.019). The results indicate that a greater proportion of progeny from more evolutionarily divergent crosses overall had higher MIC values than intermediate parental MIC values or both parental MIC values. Altogether, our findings suggest that both the individual parental strains and the hybridization process contribute to the susceptibility of progeny to fluconazole.

## 4. Discussion

In this study, we found that both parental strains and progeny can be significantly influenced by different stressors, including temperatures on their growth and oxidative and nitrosative stresses on melanin production. A range of phenotypic variations was observed between parental strains and among progeny under the tested conditions. Our results demonstrate that parental genetic divergence can impact progeny relative phenotype values, progeny phenotypic variation and plasticity, and BPH rates of progeny under some tested environmental conditions. Below we discuss our observations in more detail.

### 4.1. Aneuploidy

We determined the ploidy levels of progeny from intra-VGIII and inter-lineage crosses by FACS. Unlike their haploid parents, cryptococcal hybrids are often diploid/aneuploid [28,31,32,34,35,36,46,57]. Consistently, most progeny collected in this study were also diploid/aneuploid. Surprisingly, despite using the same selection criteria (i.e., heterozygosity at the *MAT* locus) for all progeny, only one out of 18 progeny from the intra-VGIII crosses was diploid, while over 90% of progeny from inter-lineage crosses were diploid (Table 3). These diploids were likely derived from two processes. First, they might be from the artificially terminated sexual process when we collected the dikaryotic hyphae before meiosis happened. Alternatively, they might be derived from chromosomal non-disjunction after meiosis. Due to the high similarity and compatibility between VGIII parental strains, the potentially faster mating between parental strains and more frequent chromosomal disjunction during meiosis would lower ploidy levels among progeny from the intra-VGIII crosses. In CNSC, diploidy and aneuploidy in AD hybrids are primarily caused by meiotic non-disjunction, likely due to genome divergence and genetic incompatibilities between parental strains [31,58,59]. Chromosome structural differences have been observed among the divergent lineages in the human pathogenic *Cryptococcus* [60,61,62,63]. Thus, it is tempting to speculate that the observed genome sequence and genome structure divergence between lineages facilitate the generation and maintenance of diploidy among progeny from inter-lineage crosses.

### 4.2. Mitochondrial Inheritance

In general, uniparental mitochondrial inheritance is the dominant pattern in animals, plants, and fungi [64,65]. In *C. neoformans* species complex, mitochondria inheritance of both clinical and natural AD hybrids was uniparental from the *MAT***a** parent [47,66]. Additionally, Yan and Xu demonstrated that the *MAT***α** mitochondria were selectively eliminated at an early stage during **a**-**α** mating [66]. One possibility for the uniparental inheritance of *MAT***a** parental mitochondria is that during mating, there is unidirectional migration of the *MAT***α** nucleus into the *MAT***a** cell via a conjugation tube [67]. However, mitochondria might not migrate. The newly formed dikaryotic cell would germinate to produce hyphae on the side of the mated *MAT***a** cell away from the *MAT***α** parent. Consequently, progeny developed from these hyphae would only contain the *MAT***a** mitochondria.

However, in *C. gattii* species complex, highly variable mitochondrial inheritance patterns, including from *MAT***a** parent only, *MAT***α** parent only, and recombinant mitochondrial genotypes, have been observed in previous studies and this study [68,69]. Similarly, biparental mitochondrial inheritance has been found in some other fungi, such as *Saccharomyces cerevisiae* and *Schizosaccharomyces prome* [64,70]. One potential of mitochondrial recombination in our crosses is the breakdown of genetic interactions between the parental cells governing the uniparental mitochondrial inheritance. The observation of recombinant mitochondrial genotypes only for progeny from inter-lineage VNIxVGIII and VNIVxVGIII crosses is consistent with the hypothesis of the hybrid breakdown of the mechanisms governing uniparental inheritance between divergent parents. Previously, Gyawali and Lin demonstrated that a pre-zygotic factor, Mat2, plays a crucial role in determining the mitochondrial inheritance in *C. neoformans* species complex [71]. They found that if Mat2 preactivates the pheromone pathway in the *MAT***α** parent, it can preserve the *MAT***α** mtDNA in the progeny, and vice versa. Additionally, two specific genes, *Sxi1***α** and *Sxi2***a**, were identified as essential to ensure uniparental mitochondrial inheritance. The deletion of either *Sxi1***α** and *Sxi2***a** gave rise to biparental mitochondrial inheritance, with a high proportion of progeny with recombinant mitochondrial genomes [71,72].

We examined the potential effects of mitochondria on hybrid phenotypes in this study. At most tested conditions, different mitochondrial inheritance patterns did not result in any significant phenotypic differences among progeny. However, we found that progeny with *MAT***α** mitochondria produced significantly more melanin on average than those with *MAT***a** mitochondria or recombinant mitochondrial genotypes at the non-stress condition (*p* < 0.02). This result suggests a potential adaptive significance of *MAT***α** mitochondrial inheritance. As demonstrated previously, the *MAT***α** mitochondrial inheritance and mitochondrial recombination were frequently observed under environmental stress conditions, such as ultraviolet (UV) irradiation and high temperatures, in both *C. neoformans* species complex and *C. gattii* species complex [69,73].

### 4.3. Susceptibility to the Antifungal Drug Fluconazole

Fluconazole, a triazole antifungal drug, is widely used as the first-line treatment for cryptococcal meningitis [74]. However, the frequency of drug-resistant fungal pathogens is increasing, causing severe threats to human health. Mutations in genes responsible for fluconazole resistance, such as the azole target gene *ERG11*, have been identified in fungi, including in *Candida species* and *Cryptococcus species* [75,76,77,78,79,80]. Previous studies have reported that the accumulation of aneuploidies, especially Chromosome 1 disomy, is one of the main reasons for clinical treatment failure and drug resistance in *C. neoformans* [74,81]. In this study, we found that 19 out of 55 progeny had fluconazole MIC values higher than both of their parental strains. Because all progeny were either diploid or aneuploid while parental strains were haploid, the increased fluconazole MIC values in these 19 progeny were consistent with the ploidy effects observed previously. Indeed, the only progeny (YMA136) with a lower MIC value than both parents was found to have a DNA content only slightly higher than the haploid parental strains (Figure S3). However, ploidy alone cannot explain all the observed MIC values as the remaining 36 aneuploid/diploid progeny showed no obvious advantage over haploid parental strains.

Because of the short time frame during mating and zygote analyses, the observed higher MIC values than parental strains for the 19 progeny were more likely due to gene dosage and gene expression differences rather than to de novo mutations in the progeny. For example, the gene(s) responsible for drug resistance obtained from the more resistant parent might be overexpressed in these progeny. Alternatively, epigenetic mechanisms, such as chromatin modification, could also be involved [82]. Specifically, histone acetylation has been found associated with the regulations of azole resistance in *Candida albicans* [83,84]. Also, inhibitors of histone deacetylases can impact the antifungal drug susceptibility in both *Cryptococcus neoformans* and *Aspergillus fumigatus* [85,86]. Further studies should investigate if these inhibitors impact haploid and diploid strains differently.

### 4.4. Effects of Parental Genetic Divergence on Progeny Performance

In this study, all crosses contained at least one VGIII strain due to the high fertility of VGIII strains [87,88]. Indeed, strains of VGIII lineage are genotypically highly diverse, likely due to frequent sexual recombination in nature [87,89,90,91,92]. Also, two genetically modified VGIII strains, JF101 and JF109, that have enhanced fertility were used in this study [93]. On the other hand, the fertility of strains in other lineages is relatively low. The use of one lineage (VGIII) in all crosses allows us to critically evaluate the impact of parental strain genetic divergence on progeny phenotypes.

Our analyses identified an overall negative correlation between parental genetic divergence and the growth ability of progeny at 30 °C and 37 °C. The negative correlation was likely related to genetic compatibility between the parental genomes regulating cell cycles and cell divisions. Progeny derived from inter-lineage parental strains (either evolutionarily closely related or divergent to VGIII) are likely to experience genetic incompatibility to negatively impact growth as compared to progeny of the intra-VGIII crosses. However, when progeny from intra-VGIII lineage crosses were excluded, there was a positive correlation between parental genetic distance and the growth of hybrid progeny from the inter-lineage crosses at 37 °C. At present, the exact mechanisms for the observed positive correlation are not known.

We also noticed that the presence of H_2_O_2_ led to a significant decrease in melanin production of both parents and progeny as compared to the non-stress condition. However, there was no significant difference in melanin production among the three oxidative stress levels. Previous studies have shown that actively growing *C. neoformans* cells can degrade the additional H_2_O_2_ (1 mM) within 30 min, and *C. gattii* strains are all highly tolerant to high oxidative stress (1 mM H_2_O_2_) [94,95]. Thus, the significant impact of oxidative stress on melanin production was unexpected. Surprisingly, all VGIII parental strains and progeny from the intra-VGIII crosses were not able to grow at 1 mM NaNO_2_ condition. A previous study found that strains R265 of VGII lineage and H99 of VNI lineage did not grow at 1.2 mM NaNO_2_ [96], suggesting that a higher concentration of NaNO_2_ may arrest the growth of all strains in this study. Furthermore, while many parental strains and their progeny grew at the 1 mM NaNO_2_ condition, they produced very limited amounts of melanin.

Our results revealed that progeny from evolutionarily more similar parents produced significantly more melanin under non-stress and all oxidative stresses than those from divergent parents. Progeny from intra-VGIII crosses produced significantly more melanin on average than progeny from inter-lineage crosses at low nitrosative stresses. However, most progeny from either intra-VGIII crosses or inter-lineage crosses under low and intermediate nitrosative stress, and progeny from inter-lineage crosses under high nitrosative stress produced similar amounts of melanin. Overall, our results suggest that dominant alleles are likely associated with the significant differences in responses to oxidative and nitrosative stresses in our strains, as well as the growth and melanin production under high nitrosative stress. For human pathogens, tolerance to nitrosative stress is important for their pathogenicity. Fungal pathogens (including HPC) can respond to nitrosative stress via either enzymatic defenses or non-enzymatic defenses [97]. The most common non-enzymatic defenses against nitrosative stress include melanin, mannitol, and trehalose. For example, 1,8-dihydroxynaphthalene (DHN)-melanin can protect the pathogenic fungus *Sporothrix schenckii* from nitrogen-derived radicals and macrophage-mediated killing [98]. Some proteins, such as protein kinase C (Pkc1) and isocitrate dehydrogenase (Idp1), are known to protect cell wall integrity or repair DNA damage, enabling *C. neoformans* cells to be resistant to nitrosative stress [99,100]. Similarly, transcription factor Cta4 is involved in nitrosative stress response by regulating the reactive nitrogen species (RNS) induced genes in *Candida albicans* [101]. The differential responses among lineages and their progeny to high nitrosative stress could be related to the expressions of these and/or other genes.

### 4.5. Transgressive Segregation

It has been reported that transgressive segregation occurs more frequently in plants than in animals [102]. In *Cryptococcus neoformans* species complex, Shahid et al. previously examined the vegetative fitness of serotype AD hybrids under 40 environmental conditions, including variations in temperatures, media, and fluconazole concentrations [40]. They found evidence of transgressive segregation in 39 of the 40 tested conditions. In this study, we found transgressive segregation in intra-VGIII and inter-lineage crosses under various environmental conditions in both directions. Several mechanisms have been proposed for such transgressive phenotypes in segregating populations [103,104]. Due to mitotic and meiotic recombination between parental genomes in hybrids, novel gene and allelic combinations are created, both of which could contribute to generating transgressive phenotypes in hybrid populations. Indeed, different gene and allelic combinations could generate transgressive segregations for different traits. Our observed transgressive segregations suggest the enormous capacity and potential of cryptococcal hybrids in adapting to diverse ecological niches.

### 4.6. Potential Effect of Temperature for Selecting Hybrids

In this study, we used the 37 °C temperature to select for diploid hybrids from the diverse crosses. While this process ensured that we were able to recover a large number of diploid hybrids, this temperature could potentially bias the progeny population in favor of those capable of growing at 37 °C. Indeed, the observed positive correlation between parental genetic divergence and hybrid progeny growth at 37 °C could be partly due to this selection protocol. However, for several reasons, we believe that the potential effect of the 37 °C selection temperature on the overall observed patterns is likely minor. First, as described in Results Section 3.3.1, the hybrid progeny population showed similar or even greater declines in overall growth at 37 °C (vs. at 30 °C) than the parental strains. Second, there were wide ranges of both CVs and CVi values among progeny in their growth at the 37 °C, with an overall pattern similar to those of their respective parental strains. Third, although there were slightly higher rates of both BPH and positive transgressive segregation in growth rate at 37 °C than at 30 °C, the differences were not statistically significant. Furthermore, there was also a slightly higher frequency of negative transgressive segregation at 37 °C than at 30 °C. Despite these observations, we would like to note that if the hybrid progeny selection temperature were lower (e.g., at 25 °C, which likely would require screening far more colonies to identify diploid hybrids), some of the progeny might not be able to grow at the 37 °C temperature and thus might impact our results on the measured hybrid growth abilities at this temperature [105]. In this study, all progeny selected at 37 °C grew well at both 30 °C and 37 °C. Additional experiments are needed in order to determine the potential impacts of different selection protocols on hybrid performance.

## 5. Conclusions

This study investigated the relationships between parental differences (both genetic and phenotypic) and their progeny phenotypes for three medically important traits in the human pathogenic *Cryptococcus*: growth at 37 °C, melanin production, and fluconazole susceptibility. We found several types of relationships, with progeny from each type of cross showing superior performance potential than both parents in some traits under certain conditions. There are other medically important traits in the human pathogenic *Cryptococcus*, including resistance/tolerance to other antifungal drugs, the secretion of a number of extracellular enzymes, and capsule production. At present, the relationships between parental divergence (both genetic and phenotypic) and progeny phenotype at other traits are not known and worth investigating. For capsule production, our previous research [39] and pilot experiment for this study (unpublished) revealed that, for most parental strains and progeny, their capsule size was highly variable among cells of the same sample that were grown under the same condition and measured under the same microscopic field. Often, the standard deviations were larger than the means, making most comparisons among parental strains and progeny meaningless. In this study, we analyzed highly reproducible traits, including melanin production under seven conditions, growth ability at two temperatures, and fluconazole susceptibility, all of which showed very low standard deviation when compared to the mean among repeats of the same strain. Our analyses of the three medically important traits suggest the enormous capacity and potential of cryptococcal hybrids in adapting to diverse ecological niches.

## Figures and Tables

**Table 1:** Strains of the *C. neoformans* species complex (CNSC) and *C. gatii* species complex (CGSC) were used as parents in this study.

Species Complex	Lineage	Isolate ID	Mating Type	Source
CGSC	VGI	B4495	*MAT* **a**	Clinical
		B4545	*MAT* **a**	Clinical
		WM179	*MAT* **α**	Human, CSF (cerebral spinal fluid)
		WM276	*MAT* **α**	*E. tereticornis*
		R794	*MAT* **α**	Human, CSF
		R299	*MAT* **α**	Human, CSF
	VGII	LA55	*MAT* **a**	Human, CSF
		R265	*MAT* **α**	Human, BAL
		LA61	*MAT* **α**	Human, CSF
		KB5746	*MAT* **α**	Horse
	VGIII	B4546	*MAT* **a**	Clinical
		JF109	*MAT* **a**	Lab strain
		ATCC32608	*MAT* **a**	Human, CSF
		B4544	*MAT* **α**	Clinical
		JF101	*MAT* **α**	Lab strain
		B4499	*MAT* **α**	Clinical
	VGIV	WM779	*MAT* **α**	Cheetah
CNSC	VNI	KN99**a**	*MAT* **a**	Lab strain
		KN99**α**	*MAT* **α**	Lab strain
		CDC15	*MAT* **α**	Clinical
	VNIV	JEC20	*MAT* **a**	Lab strain
		JEC21	*MAT* **α**	Lab strain

**Table 2:** Details of primers, polymerase chain reaction (PCR) protocols, and restriction enzymes that were used in this study.

Genes	Primer Sequences (5′–3′)	Amplification Conditions	Restriction Enzymes
*STE12* **α**	F: CTGAGGAATCTCAAACCAGGGA	94 °C 4 min; 35 cycles: 94 °C 45 s, 55 °C 45 s, 72 °C 1 min	NA
	R: CCAGGGCATCTAGAAACAATCG		
*STE20* **a**	F: GATCTCTCTCAGCAGGCCAC		NA
	R: AAATATCAGCTGCCCAGGTGA		
*ND2*	F: TATGATGGCCGTAGCGCTATC	94 °C 4 min; 35 cycles: 94 °C 1 min, 50 °C 30 s 72 °C 1 min	PvuII
	R: TGGTGGTACTCCTGCCATTG		
*ND4*	F: GGGAGAATTTGATTCAAGTGCAAC		SacI
	R: ATGATGTTGCATCTGGCATCATAC		
*GPD1*	F: CCACCGAACCCTTCTAGGATA	94 °C 3 min; 35 cycles: 94 °C 45 s, 63 °C 1 min, 72 °C 2 min	NA
	R: CTTCTTGGCACCTCCCTTGAG		
*LAC1*	F: AACATGTTCCCTGGGCCTGTG	94 °C 3 min; 30 cycles: 94 °C 30 s, 58 °C 30s, 72 °C 1 min	NA
	R: ATGAGAATTGAATCGCCTTGT		
*PLB1*	F: CTTCAGGCGGAGAGAGGTTT	94 °C 3 min; 30 cycles: 94 °C 45 s, 61 °C 45 s, 72 °C 1 min	NA
	R: GATTTGGCGTTGGTTTCAGT		
*IGS1*	F: ATCCTTTGCAGACGACTTGA	94 °C 3 min; 35 cycles: 94 °C 30 s, 60 °C 30 s, 72 °C 1 min	NA
	R: GTGATCAGTGCATTGCATGA		
*URA5*	F: ATGTCCTCCCA AGCCCTCGAC	94 °C 3 min; 35 cycles: 94 °C 45 s, 61 °C 1 min, 72 °C 2 min	HhaI
	R: TTAAGACCTCT GAACACCGTACTC		
*CAP59*	F: CTCTACGTCGAGCAAGTCAAG	94 °C 3 min; 35 cycles: 94 °C 30 s, 57 °C 30 s, 72 °C 1 min	HinfI
	R: TCCGCTGCACAAGTGATACCC		
*CNL06810*	F: TTAATGGACTGGGCAGATGCTCGTC	94 °C 4 min; 36 cycles: 94 °C 45 s, 55 °C 45 s, 72 °C 1 min	HhaI
	R: ATGTCTTCTCCCGCCCTTTTTGCC		
*CNI01350*	F: GAGCGACATCGTCCCTATGTGA		HinfI
	R: ACTGGTAGCAATGGCGACATG		
*CNK01700*	F: ACGCACTCTCACAGCTCCTTCG		HpyCH4IV
	R: GCAAAGCTCAGGCTCAAATCCAG		
*CNM00180*	F: GCTCAAGAACCATACCTGCTCAT		Sau96I/HpyAV
	R: GGCGGCAGGTGACTTCAGTG		
*CGND*	F: TGCGAGTCGAAGGCRGACTATGATCGTCTGATTGC	94 °C 4 min; 36 cycles: 94 °C 45 s, 60 °C 45 s, 72 °C 1 min	HinfI
	R: GCTGGATCCGTTCCTTGATAGCRGCCCACTTTGCG		
*CGNM*	F: AGCATCGTCGATGGACATCKTGGACCTTCTTCGCC		HpyCH4IV
	R: CAGAGAGCCCAGACRAAGGAGGCGAGGAACATGGC		
*ERG11*	F: CTTTGGGTGGAAAGATTTCTCAAGTCTCTGCCGAG		AluI/Sau3A
	R: GCGGCGGCAAATCCCTTTTCRTCGTGCCATCGGGC		
*CGNA*	F: AGGCCCCGAGGTTGTTGCCGARGCTGTCCGAG		AccI
	R: TCGGGGGCACCGGCGAGAGACGCAGARGGGAGGAG		
*CNB00360*	F: AGTGCTCAGAGTCTGGGGCTGG		HincII
	R: GCCATTCGCAGGGGTGGAGG		
*CNE00250*	F: TGGCGTCTCTTTGAACGCGATC		HaeII
	F: ATGGCGGAATGTCCGGCTTT		
*CNH02750*	F: TTGGATCGCTTGCTCGCGAA		XhoI
	R: AGGCCCGAGCAAAGGAATGA		

**Table 3:** Information on crosses, the genetic distance between parental strains, ploidy levels of progeny, multilocus genotypes (MLG), and minimum inhibitory concentration (MIC) values to fluconazole.

Group	Cross	*MAT*a Parent	*MAT*α Parent	Genetic Distance	Progeny ID	Ploidy	Genotype	MIC
Intra-lineage	VGIIIxVGIII	B4546 (VGIII) MIC = 1	B4544 (VGIII) MIC = 4	0.005	YMA79	A	NA	4
				0.005	YMA80	A	NA	4
				0.005	YMD81	D	NA	4
		JF109 (VGIII) MIC = 2	B4544 (VGIII) MIC = 4	0.005	YMA62	A	NA	8
				0.005	YMA63	A	NA	8
				0.005	YMA64	A	NA	8
		ATCC32608 (VGIII) MIC = 4	B4544 (VGIII) MIC = 4	0.005	YMA65	A	NA	8
				0.005	YMA66	A	NA	8
				0.005	YMA68	A	NA	8
		B4546 (VGIII) MIC = 1	JF101 (VGIII) MIC = 4	0.009	YMA73	A	NA	4
				0.009	YMA74	A	NA	4
				0.009	YMA138	A	NA	2
		JF109 (VGIII) MIC =2	JF101 (VGIII) MIC = 4	0.009	YMA102	A	NA	4
				0.009	YMA125	A	NA	4
				0.009	YMA136	A	NA	1
		ATCC32608 (VGIII) MIC = 4	JF101 (VGIII) MIC = 4	0.009	YMA77	A	NA	4
				0.009	YMA78	A	NA	4
				0.009	YMA105	A	NA	4
Inter-lineage	VGIxVGIII	B4495 (VGI) MIC = 2	B4544 (VGIII) MIC = 4	0.033	YMD53	D	MLG.21	4
				0.033	YMD90	D	MLG.19	2
				0.033	YMD96	D	MLG.12	4
		B4495 (VGI) MIC = 2	JF101 (VGIII) MIC = 4	0.038	YMD69	D	MLG.20	4
				0.038	YMA71	A	MLG.20	4
				0.038	YMD72	D	MLG.20	4
		B4545 (VGI) MIC = 2	JF101 (VGIII) MIC = 4	0.04	YMD85	D	MLG.18	4
				0.04	YMD86	D	MLG.17	4
	VGIVxVGIII	JF109 (VGIII) MIC = 2	WM779 (VGIV) MIC = 2	0.045	YMD36	D	MLG.11	2
	VGIIxVGIII	LA55 (VGII) MIC = 32	JF101 (VGIII) MIC = 4	0.128	YMD111	D	MLG.13	8
		B4546 (VGIII) MIC = 1	R265 (VGII) MIC = 4	0.135	YMD132	D	MLG.8	8
				0.135	YMD135	D	MLG.8	8
				0.135	YMD150	D	MLG.7	8
	VNIxVGIII	JF109 (VGIII) MIC = 2	KN99α (VNI) MIC = 1	0.17	YMD112	D	MLG.5	4
				0.17	YMD113	D	MLG.5	8
				0.17	YMD114	D	MLG.5	8
		B4546 (VGIII) MIC = 1	KN99α (VNI) MIC = 1	0.17	YMD29	D	MLG.5	4
				0.17	YMT33	T	MLG.5	4
		KN99a (VNI) MIC = 1	JF101 (VGIII) MIC = 4	0.171	YMD1	D	MLG.1	1
				0.171	YMD5	D	MLG.1	2
				0.171	YMD10	D	MLG.1	2
		JF109 (VGIII) MIC = 2	CDC15 (VNI) MIC = 32	0.172	YMA162	A	MLG.4	16
				0.172	YMD164	D	MLG.6	32
				0.172	YMD165	D	MLG.4	16
		B4546 (VGIII) MIC = 1	CDC15 (VNI) MIC = 32	0.172	YMD34	D	MLG.4	16
				0.172	YMD83	D	MLG.2	16
				0.172	YMT98	T	MLG.3	4
	VNIVxVGIII	JF109 (VGIII) MIC = 2	JEC21 (VNIV) MIC = 1	0.172	YMD87	D	MLG.9	4
		B4546 (VGIII) MIC = 1	JEC21 (VNIV) MIC = 1	0.172	YMD88	D	MLG.10	4
				0.172	YMD89	D	MLG.9	4
		JEC20 (VNIV) MIC = 1	JF101 (VGIII) MIC = 4	0.173	YMD16	D	MLG.15	4
				0.173	YMD17	D	MLG.16	4
		JEC20 (VNIV) MIC = 1	B4544 (VGIII) MIC = 4	0.171	YMD11	D	MLG.14	8
				0.171	YMD12	D	MLG.22	8
		ATCC32608 (VGIII) MIC = 4	JEC21 (VNIV) MIC = 1	0.171	YMD25	D	MLG.10	4
				0.171	YMD26	D	MLG.10	4
				0.171	YMD27	D	MLG.1	4

A: aneuploidy; D: diploidy; T: triploidy. NA: genotype was not able to be determined.

## Data Availability

Not applicable.

## Supplementary Materials

The following are available online at https://www.mdpi.com/article/10.3390/jof7040299/s1, Figure S1: Effects of oxidative and nitrosative stress on average melanin production of parental strains, Figure S2: Effects of oxidative and nitrosative stress on average melanin production of progeny, Figure S3: FACS profile of YMA136 comparing to the haploid control and diploid control, Table S1: Information on genotyping and mitochondrial inheritance, Table S2: Coefficient of variation (CV) in growth and melanin under various environmental conditions, Table S3: Better parent heterosis (BPH) and transgressive segregation found under various environmental conditions, Table S4: Relationships between oxidative stresses and nitrosative stresses.
