# Supplementary material for: What Are the Best Parents for Hybrid Progeny? An Investigation into the Human Pathogenic Fungus Cryptococcus
Source: J Fungi (Basel). 2021 Apr 15;7(4):299. doi: 10.3390/jof7040299 (PMC8071107; doi:10.3390/jof7040299)
Supplement: Supplementary file 1 [file jof-07-00299-s001.zip › Supplementary file/Supplementary figures.docx]

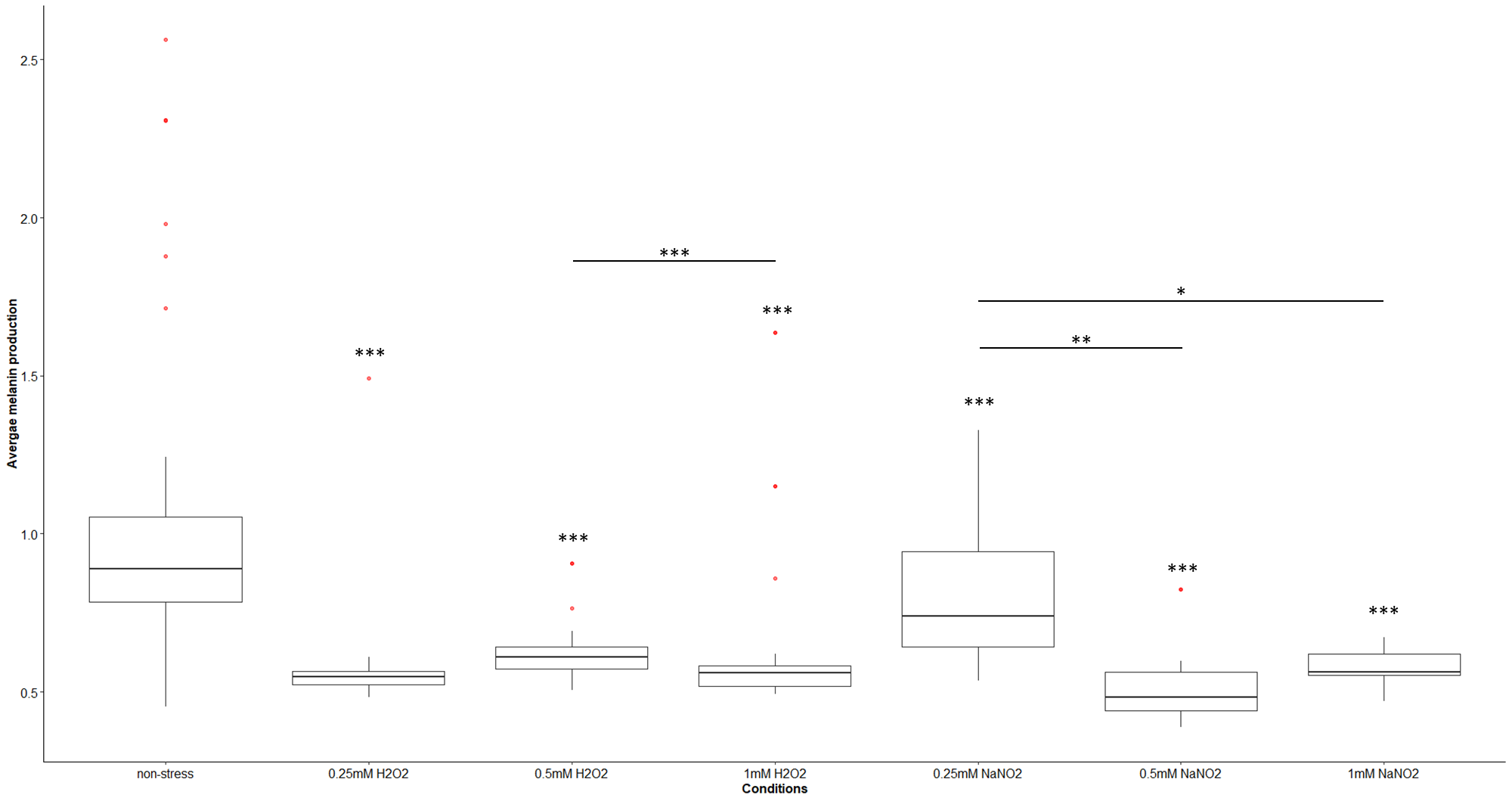


**Figure S1**. Effects of oxidative and nitrosative stress on average melanin production of parental strains. At high nitrosative stress, we excluded those parental strains that did not grow. Pairwise comparisons were performed between stresses. Red dots represent outliers. * indicates *p* values < 0.05; ** indicates *p* values < 0.005; *** indicates *p* values < 0.001.


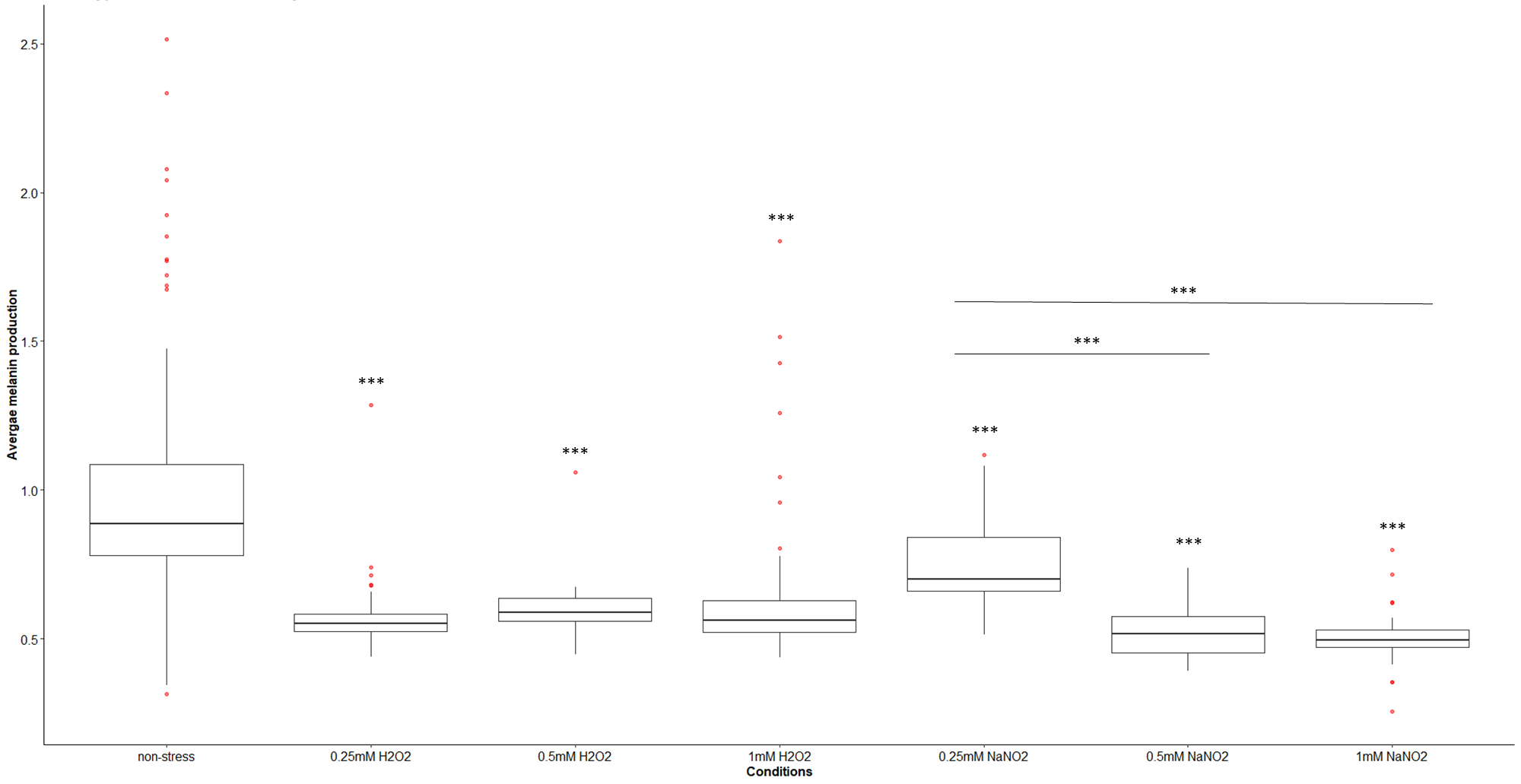


Figure S2. Effects of oxidative and nitrosative stress on average melanin production of progeny. Seven environmental conditions, including non-stress, oxidative stress (low, intermediate, and high), and nitrosative stress conditions (low, intermediate, and high) were tested in this study. At high nitrosative stress, we excluded progeny that did not grow. Red dots represent outliers. *** indicates that the *p*-value is less than 0.001.


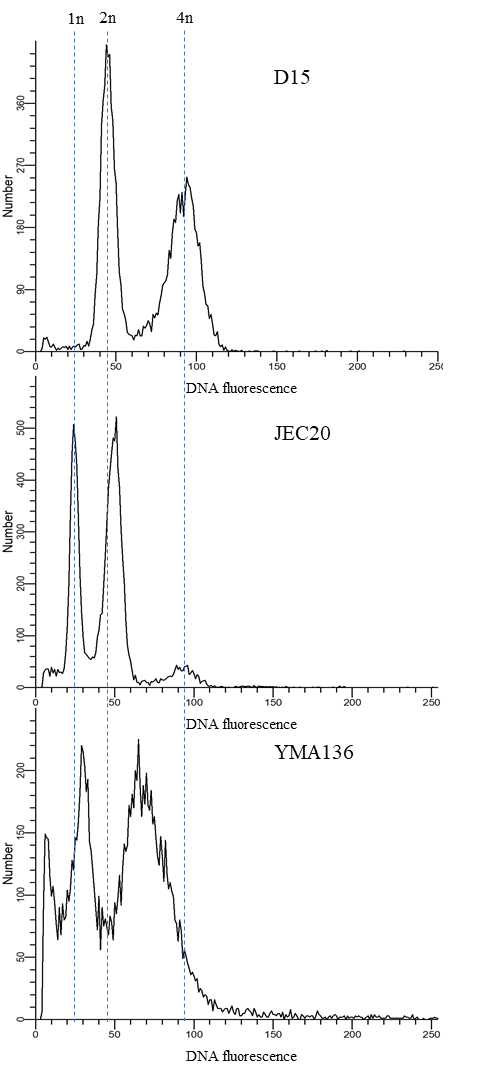


**Figure S3**. FACS profile of YMA136 comparing to the haploid control and diploid control.
